# UVB-Induced Cell Death Signaling Is Associated with G1-S Progression and Transcription Inhibition in Primary Human Fibroblasts

**DOI:** 10.1371/journal.pone.0076936

**Published:** 2013-10-14

**Authors:** Tatiana Grohmann Ortolan, Carlos Frederico M. Menck

**Affiliations:** Department of Microbiology, Institute of Biomedical Sciences, University of São Paulo, Sao Paulo, SP, Brazil; German Cancer Research Center, Germany

## Abstract

DNA damage induced by ultraviolet (UV) radiation can be removed by nucleotide excision repair through two sub-pathways, one general (GGR) and the other specific for transcribed DNA (TCR), and the processing of unrepaired lesions trigger signals that may lead to cell death. These signals involve the tumor suppressor p53 protein, a central regulator of cell responses to DNA damage, and the E3 ubiquitin ligase Mdm2, that forms a feedback regulatory loop with p53. The involvement of cell cycle and transcription on the signaling to apoptosis was investigated in UVB-irradiated synchronized, DNA repair proficient, CS-B (TCR-deficient) and XP-C (GGR-deficient) primary human fibroblasts. Cells were irradiated in the G1 phase of the cell cycle, with two doses with equivalent levels of apoptosis (low and high), defined for each cell line. In the three cell lines, the low doses of UVB caused only a transient delay in progression to the S phase, whereas the high doses induced permanent cell cycle arrest. However, while accumulation of Mdm2 correlated well with the recovery from transcription inhibition at the low doses for normal and CS-B fibroblasts, for XP-C cells this protein was shown to be accumulated even at UVB doses that induced high levels of apoptosis. Thus, UVB-induced accumulation of Mdm2 is critical for counteracting p53 activation and apoptosis avoidance, but its effect is limited due to transcription inhibition. However, in the case of XP-C cells, an excess of unrepaired DNA damage would be sufficient to block S phase progression, which would signal to apoptosis, independent of Mdm2 accumulation. The data clearly discriminate DNA damage signals that lead to cell death, depending on the presence of UVB-induced DNA damage in replicating or transcribing regions.

## Introduction

Ultraviolet (UV) solar radiation is comprised of three wavelengths, UVA (320–400 nm), UVB (290–320 nm) and UVC (100–290 nm). The first two, by inducing cellular DNA damage, constitute important environmental carcinogens. Wavelengths below 290 nm, i.e., those corresponding to the UVC portion, are efficiently absorbed by the atmospheric ozone layer. The most abundant UV induced DNA lesions are cyclobutane pyrimidine dimers (CPDs) and pyrimidine (6–4) pyrimidone photoproducts (6–4 PPs) [1], [2].

Nucleotide excision repair (NER) is the main mechanism involved in the removal of bulky helix distorting lesions, such as those induced by UV. This type of damage interferes with both normal DNA base pairing, and replication and transcription processes. If not removed, it may lead to cytotoxicity and mutagenesis. NER, a complex process involving the participation of around 30 proteins in human cells [3], [4], operates through two sub-pathways, viz., transcription-coupled repair (TCR), which is selective for lesions in the transcribed strand of active genes, and specific for damage that blocks elongation of RNA-polymerase II, and global genome repair (GGR), active with lesions throughout the genome, including silenced regions and non-transcribed strands of active genes [5], [6].

Mutations in those genes involved in NER can cause rare human hereditary diseases, such as Xeroderma Pigmentosum (XP) and the Cockayne Syndrome (CS). The genes involved in complementation groups A, B, D, F and G are necessary for both NER sub-pathways, whereas XP-C and XP-E cells are deficient in GGR but proficient in TCR. The genes involved in the two complementation groups in the Cockayne Syndrome, viz., CS-A and CS-B, are required for TCR only [7], [8].

In addition to DNA repair, cells dispose of several mechanisms when dealing with DNA damage [9], [10]. The tumor suppressor protein p53, which plays a central role in the regulation of cell response to different forms of stress, including DNA damage [11], is capable of stimulating DNA repair, promoting delays in cell-cycle progression, and inducing apoptosis and senescence, thereby regulating crucial processes used by cells to respond to genotoxic stress. The tetrameric form of p53 can bind to specific DNA sequence elements and activate the transcription of hundreds of target genes [12]–[14]. The capacity of p53 in delaying the cell cycle appears to be mediated by only a few genes, such as p21, which contributes to G1/S arrest by inhibiting the cyclin-dependent kinase complexes that promote S phase entrance [12], [15]. The transactivation of pro-apoptotic genes is one of the functions of p53 in the induction of cell death, although other transcription-independent functions also appear to contribute to the process [16]–[18].

In the absence of cell stress, p53, an unstable protein with a short half-life, is constantly subjected to degradation by the ubiquitin-proteasome system. It is targeted for degradation by the ubiquitin ligase Mdm2, which also inhibits p53 transcriptional activity through direct interaction with its amino-terminal transactivation domain. Moreover, Mdm2, as a transcriptional target of p53, together form a regulatory loop that controls the levels of both proteins [12], [13], [19].

In response to cell stress, p53 may be stabilized due to its diminished degradation, which is controlled mainly in the context of its interaction with Mdm2 [20]. This interaction is affected by post-translational modifications in both proteins, besides the effect of other regulatory pathways that may attenuate or stimulate p53 activation [21]. Mdm2 is also capable of stimulating its own degradation, thereby contributing to the complete activation of p53 [12], [22], [23].

TCR-deficient human fibroblasts accumulate p53 and induce apoptosis at lower UV doses than cells proficient in this NER sub-pathway, thereby indicating that lesions present in the transcribed regions of the genome serve as signals for the stabilization of p53 and apoptosis [24]–[26]. GGR-deficient XP-C fibroblasts present a similar dose-response in relation to the accumulation of p53 and RNA synthesis, when compared to normal fibroblasts [25]. However, from studies with primary human fibroblasts [27] and CHO cells [28], [29], it was inferred that UV-induced apoptosis could be associated with cell progression through the S phase. In fact, signaling resulting from the encounter of lesions during replication as well as the generation of DNA double-stranded breaks, due to the replication of non-repaired damage or due to the destabilization and collapse of replication forks in front of persistent lesions during S phase, contribute to explain the induction of cell death in UV-irradiated cells [30].

While UV-induced lesions interfere with both transcription and replication, the specific contribution of each process to apoptosis induction remains uncertain. Nonetheless, the idea that persistent signaling from DNA lesions leads to apoptosis would, in principle, be applicable to lesions that block either transcription or replication. This idea fits into a model where the cell fate after genotoxic stress is determined by the capacity of cells to end the apoptotic response mediated by p53 before pro-apoptotic genes have been transcribed and expressed to a threshold level in which the apoptotic process is irreversibly induced. Consistent with this model, the absence of Mdm2 induction in human fibroblasts deficient for TCR, was associated with apoptosis after UV [31], thus compatible with the negative role of Mdm2 in the p53 function. Although in that study the same UVC doses for repair proficient and deficient cells were used, it is possible to speculate that Mdm2 induction would be dependent on the UV dose, and that the same mechanism operates for both repair deficient and proficient cells, the outcome being simply defined by the extent of unrepaired DNA damage, and its resultant intracellular signaling. In fact, both p53 and Mdm2 regulation, as well as their interaction, were shown to be dose-dependent in human fibroblasts [32].

This study addresses the responses of synchronized primary human fibroblasts with different repair capacities to the irradiation with the physiologically relevant UVB wavelengths and relates them to the induction of Mdm2 protein. Our observations extend to cells with different repair proficiencies a mechanism in which termination of p53 activation would be critical for G1/S progression and avoidance of apoptosis in G1-irradiated cells, and strengthens the role of Mdm2 as an important marker associated with recovery from UVB-induced transcriptional stress in human skin fibroblasts. These observations also stress that even after recovery from transcriptional arrest, the presence of unrepaired DNA lesions can lead to cell cycle arrest, possibly due to DNA synthesis blockage and apoptosis, a pathway that is enhanced in the case of XP-C deficient fibroblasts.

## Methods

### Ethics Statement

This work was performed with primary human cells in culture, part of a biorepository approved by the Ethical Committee for the Research with Human Samples, of the Institute of Biomedical Sciences, University of São Paulo.

### Cell culture

Primary human fibroblasts were obtained from skin biopsies of a normal individual (FHN) [33], a Xeroderma Pigmentosum group C patient (XP17VI) [34] and a Cockayne Syndrome group B patient (GM00739, Coriell Cell Repositories). The cells, kindly provided by Dr. Alain Sarasin (IGR, Villejuif, France) and Dr. Claudimara Lofti, were cultivated in a Dulbeccós Modified Eagle Medium (LGC Biotecnologia, São Paulo, SP, Brazil) supplemented with 15% fetal bovine serum (FBS) (Cultilab, Campinas, SP, Brazil), 100 U/ml of penicillin G sodium, 100 μg/ml of streptomycin and 0.25 mg/ml of amphotericin B (Life Technologies, Carlsbad, CA). They were grown at 37°C, in a humidified, 5% CO_2_ atmosphere.

### Synchronization in the G1 phase of the cell cycle

Cell cultures, confluent for 3 to 5 days, were used to seed plates. Irradiation took place twelve hours later, when cell adherence was complete. At this point, and as confirmed by flow cytometry, around 90% had already entered the G1 phase of the cell cycle. Nonetheless, up to 18 hours after plating, none were detected in the S phase.

### Irradiation with UVB

Cells were irradiated by using a Vilber Lourmat VL-215MC apparatus with a 15 W lamp emitting predominantly at 312 nm at a dose rate of 5 J/m^2^s. Intensity was measured with a Vilber-Lourmat VLX3W dosimeter coupled to a CX-312 probe (Marne la Valle, France). Irradiation was in PBS within enclosed plates, to so block any residual UVC incidence. Afterwards and prior to collection, cells were incubated in fresh culture media over different time periods.

### Flow cytometry for sub-G1 and cell cycle analysis

Seventy-two hours after UVB irradiation, adherent cells, collected by trypsinization and combined with floating cells, were suspended in PBS/70% ethanol and stored at −20°C. They were then stained with 50 µg/ml of propidium iodide (PI) for one hour in the presence of 40 μg/ml of RNase A, prior to analysis by flow cytometry, using Guava EasyCyte Plus (GE). Results were analyzed with WinMDI 2.8 and ModFit LT softwares.

### Morphologial detection of apoptosis and necrosis

Cells were collected by trypsinization together with floating cells and suspended in 30–50 µl of PBS. Slides were prepared with 8 µl of the cell suspension and 2 µl of a dye mix containing 0.1 mg/ml of Hoescht 33342, 0.25 mg/ml of PI and 0.5 mg/ml of fluorescein diacetate. A Zeiss Axiovert 200 microscope was used for visualization. At least 500 cells were classified and quantified as viable, early apoptotic, late apoptotic and necrotic, according to cytomorphological and membrane permeability criteria [35].

### Determination of RNA synthesis

RNA synthesis was defined through pulse-labeling of nascent RNAs with 10 μCi/ml of ^3^H-uridine (Perkin Elmer) in DMEM containing 3% of dialyzed FBS for 1 hour at 37°C. Cells were trypsinized and lysed with a solution containing 20 mM Tris-Cl, 300 mM NaCl, 2 mM EDTA, 1% SDS and 200 μg/ml of Proteinase K for 5 minutes at 37°C. The lysates were applied to thick filter paper, fixed with TCA 15% and washed with ethanol, followed by measurement of ^3^H-uridine incorporation by liquid scintillation. The amount of DNA, as measured by absorbance at 260 nm, was used to normalize the samples. Normalized values were then divided by the results obtained for the non-irradiated controls for each time point to determine the percentage of transcriptional recovery.

### Western blots

Cells were collected by trypsinization together with floating cells and then lysed with a RIPA buffer containing protease inhibitors. Lysates (50–100 µg) were separated on 7.5% or 12% denaturing gels and transferred to nitrocellulose membranes. The antibodies used were cleaved caspase-3 (Cell Signaling Technology), p53 (DO-7, DAKO), Mdm2 (SMP14, Santa Cruz Biotechnology), actin (I-19, Santa Cruz Biotechnology), beta-tubulin (H-235, Santa Cruz Biotechnology), and HRP-labeled secondary antibodies from Molecular Probes. Reactive bands were visualized using a chemiluminescent reagent from Millipore, followed by capture in Image Quant photodocumentation equipment (GE).

## Results

### UVB induces human fibroblast apoptosis at a linear dose-response rate

**Figure 1:**
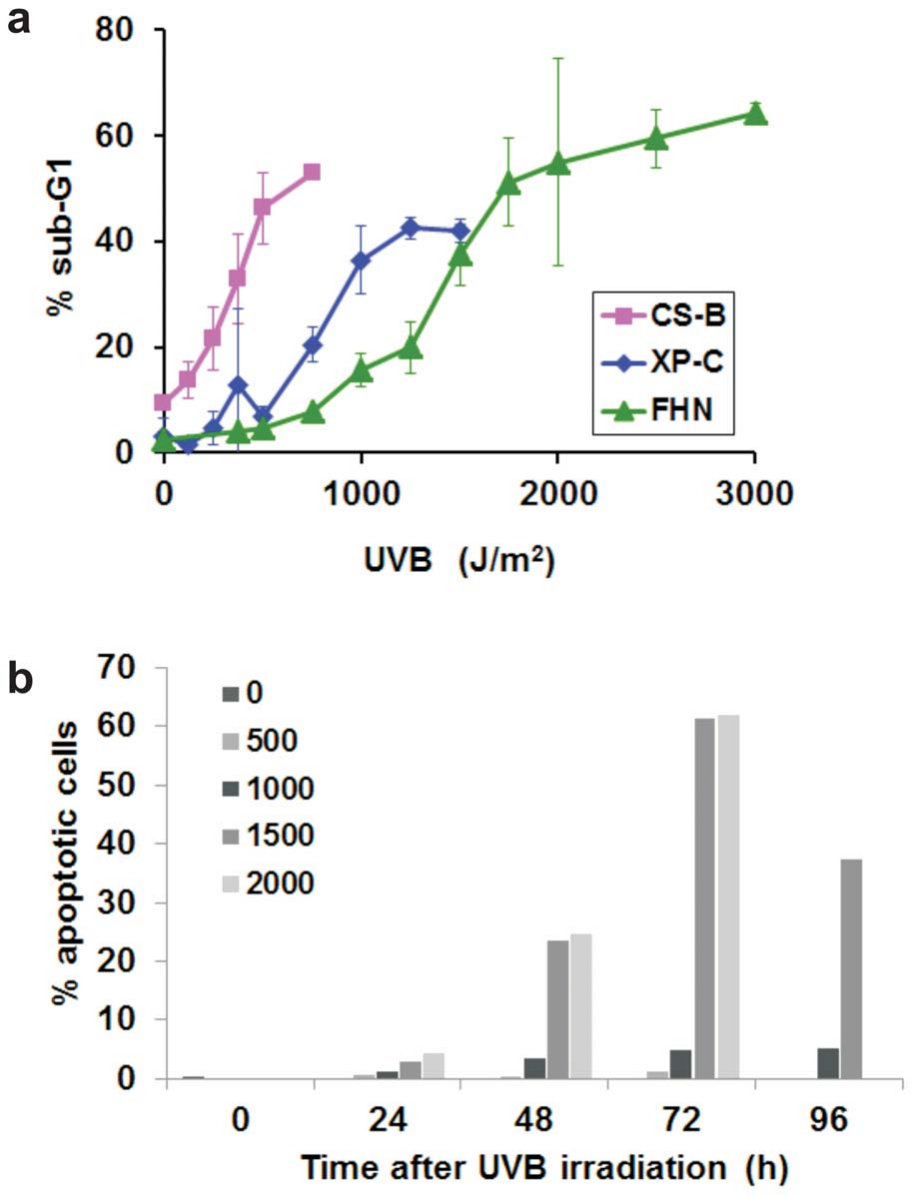
UVB induced apoptosis in FHN, XP-C and CS-B fibroblasts: a) Cells were synchronized in G1, irradiated with steadily increasing doses of UVB, and collected 72 hours after the irradiation. Apoptosis rates were determined by sub-G1 quantification through flow cytometry. b) Cytomorphological analysis of FHN fibroblasts was to determine the percentage of apoptotic cells.

The apoptosis rates for normal (FHN), GGR-deficient (XP-C) and TCR-deficient (CS-B) primary human fibroblasts were determined, 72 hours after irradiation with increasing doses of UVB, by flow cytometry-quantification of the cellular sub-population with sub-G1 DNA content. Synchronized cells were irradiated when predominantly in the G1 phase of the cell cycle. The results are shown in Figure 1a, where it is possible to observe that the central part of the individual curves obtained present an approximately linear dose-response rate. For the three cell lines, the apoptosis levels reached a plateau, where levels of apoptosis were saturated with increasing doses of UVB. Two doses with equivalent toxicity were defined for each cell line, one considered as “low dose”, with at least 5% increased apoptosis in comparison with the non-irradiated controls, and the other as “high dose” chosen within the early plateau regions of the curves for each cell line. The defined doses resulted in apoptosis rates within the range 10–20% for low doses, and 40–60% for high ones. Table 1 indicates the low and high dose ranges of UVB defined for each cell line based on the above.

**Table 1:** UVB doses defined as low or high based on the levels of apoptosis (sub-G1) induce in each cell line (based on Figure 1a).

Fibroblasts	Low dose (J/m^2^)	High dose (J/m^2^)
**CS-B**	125–250	500
**XP-C**	375–500	750–1000
**FHN**	750–1000	2000

Microscopic assays, for examining alterations in cytomorphology and membrane integrity, also corroborated cell death by apoptosis in FHN cells (Figure 1b). The data confirm the time interval of 72 hours as being adequate for the satisfactory differentiation between low and high doses of UVB, since the difference in apoptosis was much more pronounced than with the 48 hour time interval, whereas at the 96 hour point, the counting of apoptotic events was compromised by cell lysis in late apoptosis. The results were also consistent with the death rates shown in Figure 1a for FHN fibroblasts.

### Normal and NER-deficient fibroblasts show cell-cycle blockage after equivalent high UVB doses

**Figure 2:**
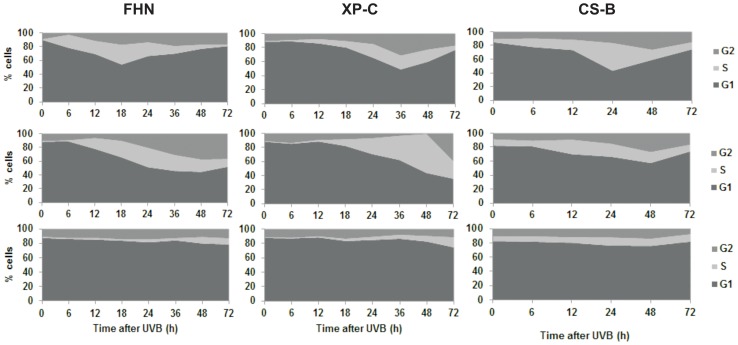
Effect of the irradiation of FHN, XP-C and CS-B fibroblasts with low or high UVB doses during cell cycle progression: Cells were synchronized in G1, irradiated with different doses of UVB, and collected at the indicated times after irradiation. Fluorescence histograms were obtained for each condition after PI staining and flow cytometry. The proportion of cells in the G1, S and G2 phases of the cell cycle were analyzed and quantified for each histogram using ModFit LT. The results obtained were combined to generate the graphs presented. UVB doses used were: upper panels – non-irradiated controls; middle panels – low doses (750, 375 and 125 J/m^2^ for FHN, XP-C and CS-B respectively); lower panels – high doses (2,000, 750 and 500 J/m^2^ for FHN, XP-C and CS-B respectively).

Cell cycle progression was examined after irradiation of FHN, XP-C and CS-B fibroblasts with the defined low and high doses of UVB. Cells were irradiated in the G1 phase of the cell cycle, collected at different periods, and then submitted to flow cytometry. The histograms so generated were analyzed with ModFit LT software, to determine the proportion of cells in G1, S and G2/M for each time point and dose. The results obtained are compiled in Figure 2. For both NER proficient and deficient cells, there were delays in progression to the S phase after low UVB doses, in comparison with the non-irradiated controls, as shown by the delayed increase over time, in the percentage of S phase cells. Both non-irradiated fibroblasts and fibroblasts irradiated with low doses of UVB showed a decrease in G1 cells over time, with a concomitant increase in the proportion of S phase and, at later periods, G2 phase cells. With time, the amount of G1 cells gradually increased, due to cell division, as well as growth-contact inhibition, which normally causes an accumulation of cells at this phase. The one exception was XP-C fibroblasts irradiated with the low dose of UVB, since for which, the proportion of G1 cells kept decreasing up to the longest time tested, i.e., 72 hours. Up to 48 hours after irradiation, this was accompanied by a steady increase in S phase cells, thereby inferring the pronounced difficulty for XP-C cell progression through the S phase to G2, and subsequently, to cell division. Thus, for these UVB doses that induce only low levels of apoptosis, the cells are capable to progress their cell cycle, despite of a certain delay. Interestingly, after the high doses of UVB, the distribution of cells in the different phases of the cell cycle remained practically constant, an indication of prolonged cell cycle arrest in G1 phase, for the three cell lines. As these high doses represent the early plateau of apoptosis, the result indicates the cells are unable to progress through S phase triggering cell death.

### Recovery of transcription after UVB in normal and NER-deficient fibroblasts

**Figure 3:**
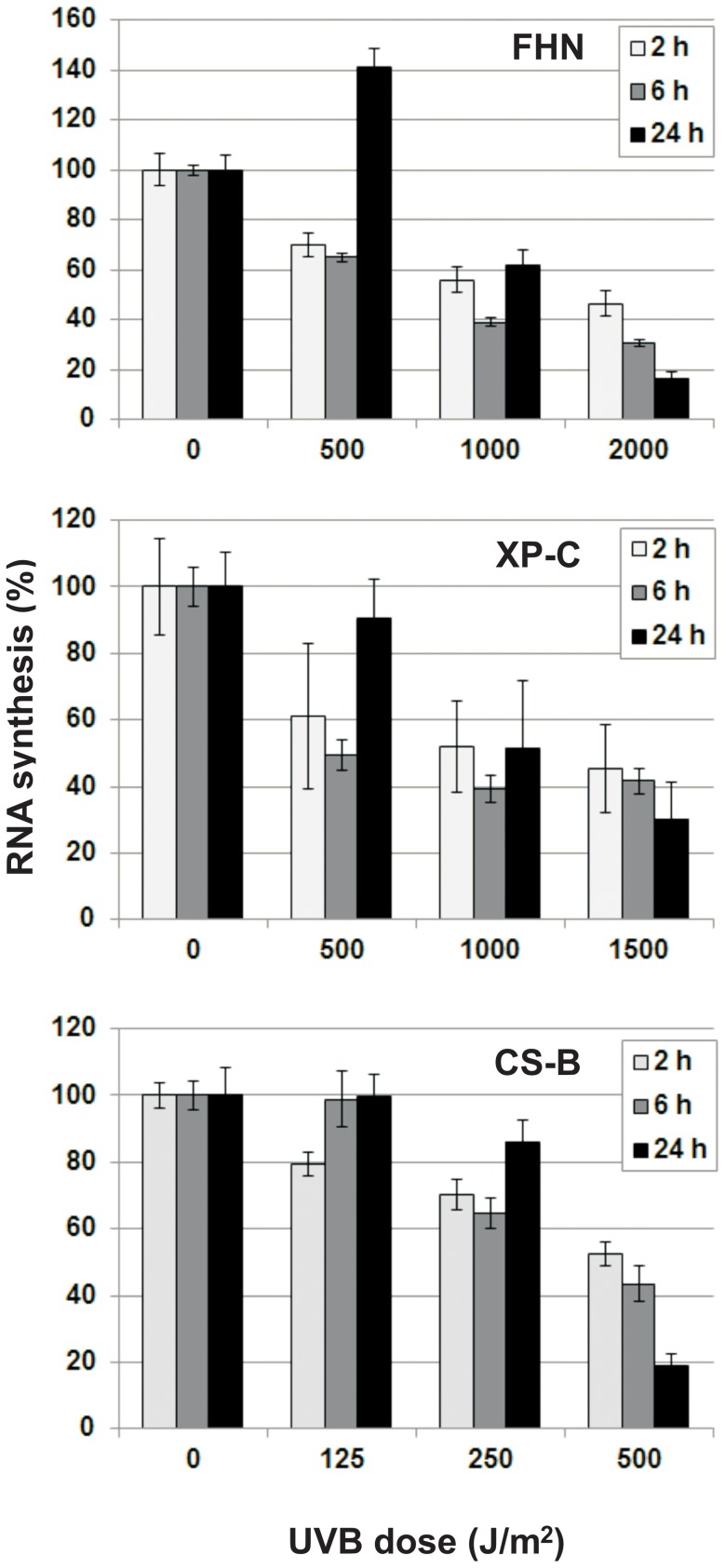
Recovery of transcription in FHN, XP-C and CS-B fibroblasts after irradiation with low and high UVB doses: Cells were synchronized in G1, irradiated with different UVB doses, and labeled with tritiated uridine for 1-irradiated controls for each time-point.

The capacity of FHN, XP-C and CS-B fibroblasts to recover from transcription inhibition was checked up to 24 hours after irradiation with low and high doses of UVB (Figure 3). FHN and CS-B fibroblasts presented transcriptional recovery after the application of low doses (500 and 1,000 J/m^2^ for FHN and 125 and 250 J/m^2^ for CS-B), but not after high doses (2,000 J/m^2^ and 500 J/m^2^, respectively). As to XP-C cells, a very similar pattern to that of DNA-repair proficient cells was observed, when analyzed in terms of absolute UVB-dose irradiation. Notwithstanding, based on apoptosis rates, in the case of UVB-doses with similar relative killing efficiency, transcription recovery was observed at both the low and high doses (500 and 1,000 J/m^2^), but not after a still higher dose (1,500 J/m^2^), up to 24 hours post-irradiation.

### Accumulation of p53 and Mdm2 after UVB damage

**Figure 4:**
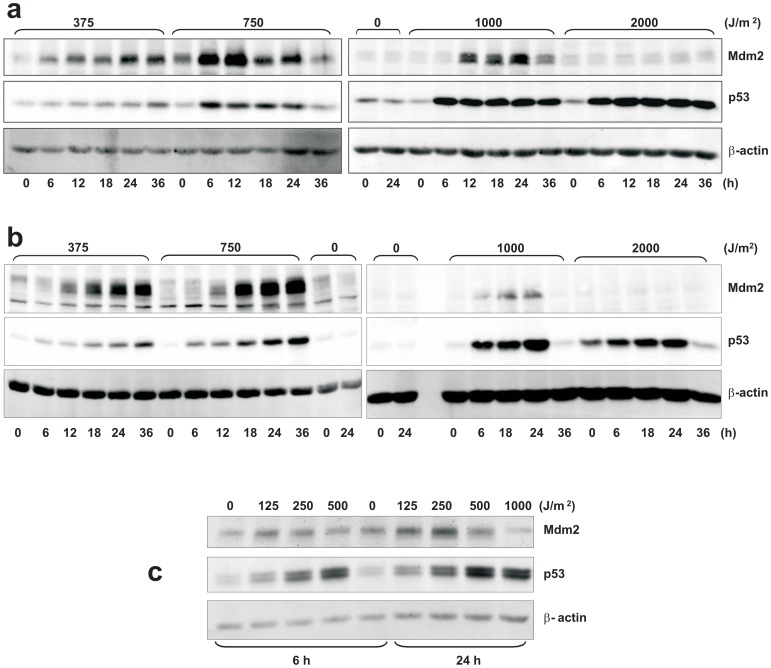
Western blot detection of p53 and Mdm2 proteins: FHN (a), XP-C (b) and CS-B (c) fibroblasts were irradiated with the indicated doses of UVB and collected at the time intervals shown. Beta-actin was used as loading control.

p53 induction in FHN, XP-C and CS-B fibroblasts after UVB irradiation was confirmed by western-blot. As expected, induction generally became more pronounced with the increase in UVB dose (Figure 4). As to Mdm2 levels, more detailed temporal data were collected for FHN and XP-C cells (Figure 4, panels a and b). The results were strikingly similar for both cell lines, despite differences in repair capacities and UVB sensitivity. On comparing doses of 375 and 750 J/m^2^, the more efficient p53 induction at the higher dose could be associated with increased Mdm2 levels. Accumulation of Mdm2 occurred after a dose of 1,000 J/m^2^, but not after that of 2,000 J/m^2^. As to CS-B cells, Mdm2 induction was observed 6 hours after irradiation with 125 J/m^2^, and 24 hours after doses of 125 and 250 J/m^2^. Therefore, in the case of FHN and CS-B, a correlation certainly exists between Mdm2 accumulation and cell survival, since Mdm2 increased-protein levels were absent at UVB doses defined as being associated with high levels of cell death by apoptosis. On the other hand, XP-C cells did not present that correlation, in the same way they had not in the experiments dealing with transcriptional recovery. However, on considering absolute doses, there appears to be a similar response between FHN and XP-C. Thus, at least for XP-C cells, there was every indication of apoptosis induction, despite the high levels of Mdm2 expression (e.g., after 750 J/m^2^ – Figure 4b).

**Figure 5:**
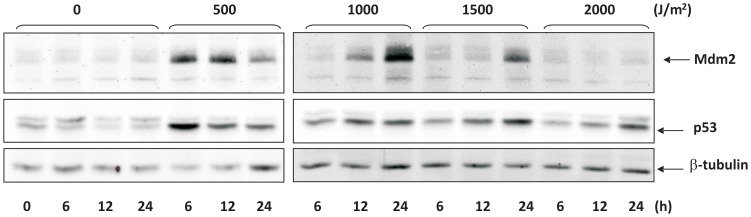
Kinetics of protein levels of Mdm2 and p53 in FHN fibroblasts irradiated with increasing doses of UVB (as indicated). Beta-tubulin was used as loading control.

To better understand the timing of Mdm2 induction in human cells, up to 24 hours after irradiation, FHN fibroblasts were irradiated with consecutively increasing doses of UVB and checked for Mdm2 levels. Clearly, a retarded increase in these levels was observed as higher UVB doses were used, in comparison with the lowest dose of 500 J/m^2^ tested, after which the accumulation of Mdm2 was evident, 6 hours post-irradiation (Figure 5).

## Discussion

Dermal fibroblasts obtained through skin biopsies from healthy individuals and patients with XP and CS syndromes, are particularly important in investigating factors involved in apoptosis signaling. Primary fibroblasts have the additional advantage of incorporating physiological levels and regulation of p53 proteins, as well as the other signal-transduction pathways involved in cell response to DNA damage. Even so, in the past, only scarce data correlating apoptosis in primary human fibroblasts with physiological UV doses were available. This is particularly so for the biologically relevant UVB component of solar radiation, since UVC wavelengths emitted by germicidal lamps were often employed. In the available studies, a wide range of doses (50 to 3,500 J/m^2^) was used [32], [36]–[38]. Herein, it was considered crucial to define an interval of UVB doses, where cell death by apoptosis would be either very low, or high enough but still within the linear increase range, in order to explore those cell mechanisms liable for detecting DNA damage, and translating the acquired information into the various forms of cell response. Furthermore, irradiation of cells in the G1 phase facilitated both studying the effects of DNA-lesion induction in the absence of DNA replication, and following cell progression into the S phase.

As expected, normal fibroblasts were more resistant to UVB irradiation than NER deficient XP-C and CS-B cells (Figure 1a). When compared with XP-C cells, the greater sensitivity of CS-B cells to UVB-induced apoptosis indicated the stronger contribution of lesions in the transcribed portions of the genome (and thus normally corrected by TCR) to apoptosis signaling, whereas XP-C fibroblasts were more sensitive to cell death by apoptosis than normal ones, a clear contribution of lesions in the non-transcribed and silenced regions of the genome to UVB-irradiation induced cytotoxicity.

CS-B fibroblasts presenting much weaker recovery from RNA synthesis inhibition after UVB irradiation than XP-C and DNA repair proficient fibroblasts, was consistent with the role of TCR in the removal of transcription blocking lesions. Interestingly, RNA synthesis recovery was still efficient at low UVB doses, which induced low levels of apoptosis (Figure 3). The kinetics for recovery from transcriptional blockage was amazingly similar in TCR-proficient normal and XP-C cells, in spite of the much higher sensitivity in the latter. For example, at a UVB dose of 1,000 J/m^2^, XP-C cells were still capable of partially recovering RNA synthesis, as were FHNs, even though in the former (XP-C cells), the levels of apoptosis and permanent cell cycle arrest were high. Cell cycle arrest and cell sensitivity, at UVB doses that do not elicit a strong transcriptional blockage, probably reflect a significant obstruction of DNA replication caused by lesions not removed, due to deficient GGR. From this perspective, the permanent arrest of XP-C fibroblasts irradiated with the high UVB dose of 750 J/m^2^ (Figure 2) could be explained by initiation of the S phase through the removal of transcription-blocking lesions by TCR, followed by difficult progression through this phase, due to the excess of replication-blocking lesions, thereby generating G1/S arrest. This cell cycle blockage normally occurs at a UVB dose that eventually leads to cell death.

A correlation between recovery from transcription inhibition (Figure 3) and accumulation of Mdm2 (Figures 4 and 5) was observed for all the UVB doses tested, independent of cell DNA-repair capacity. In CS-B cells, Mdm2 accumulation occurred at low UVB doses, thus consistent with the low capacity for recovery from transcription inhibition. Accumulation of this protein was similar for both XP-C and FHN cells. Transduction pathways that receive inputs from DNA damage signaling probably control both *MDM2* gene expression and Mdm2 protein stability. Once the triggering signal is terminated, there is a rise in Mdm2 protein levels and general transcription is resumed. Once stabilized, Mdm2 can counteract p53 activation and hinder progression to the apoptotic threshold through transcription of pro-apoptotic genes. The extended time interval for accumulation of Mdm2 after irradiation with increasing doses of UVB (Figure 5) diminished the window of opportunity for inactivation of p53-mediated apoptotic response, thereby compromising cell survival.

As far as we know, this study is the first to characterize the cell cycle progression of primary human fibroblasts after UVB irradiation in the G1 phase. It is interesting to observe that the three cell lines tested presented similar responses, regardless of their different repair capacities, i.e. delays in cell-cycle progression after low doses of UVB and permanent arrest after high doses (Figure 2). These observations with human cells are consistent with experiments with hamster cells in culture [39], in which irradiation of CHO cells synchronized in G1 with UVC resulted in dose-dependent delays for entering the S phase. Furthermore, prolonged blockage in G1 leading to apoptosis was observed in a subpopulation of hamster cells under similar conditions [40]. Here, apoptosis induction was observed in arrested cells, coincident with the G1 phase in which they were irradiated. In CHO isogenic cell lines synchronized in G1 and irradiated with UVC, both temporary and prolonged arrests in G1 in TCR-deficient cells occurred after lower UV doses, thereby inferring that the persistence of damage in transcribed regions of the genome inhibits progression from G1 to S [40].

In this work with human cells, cell cycle delays and arrests were also observed with the lowest UVB doses in TCR-deficient CS-B fibroblasts. Therefore, the removal of transcription blocking lesions might be the determinant factor in G1/S progression after irradiation with UVB in G1. In this sense, it is also possible that a more direct association exists between Mdm2 accumulation and recovery from G1 arrest after UVB irradiation in human fibroblasts. It was shown that Mdm2 and its analog Mdm4, through mediation by proteasome in the G1 phase and early S, interact directly with p21 thereby regulating its degradation in an independent, although cooperative, way [41]. Thus, accumulation of Mdm2 is probably involved in the release from G1 arrest caused by UV-induced p53 transactivation of p21.

In the case of XP-C cells, apoptosis induction occurred at doses where Mdm2 accumulated, thus demonstrating this is not sufficient for cell-life maintenance. In fact, the stronger correlation of apoptosis with permanent arrest in G1/S was probably due to complete DNA-synthesis blockage, thus consistent with previous works describing the effects of DNA replication in cell death induction by UV [1], [29], [30], [34].

## Conclusions

Our data, besides showing the strong correlation between Mdm2 accumulation and recovery from transcription inhibition in primary human fibroblasts irradiated with UVB in G1, point to the removal of damage in transcribed regions of the genome as the determining factor towards this correlation. Thus, the tumor suppressor protein p53 and its binding partner Mdm2 form a critical regulatory node in response to UVB-induced stress. Signaling from DNA damage that block transcription contributes to p53-induction, which then transactivates Mdm2. In TCR deficient cells (CS-B), this transcription blockage and thus lack of Mdm2 accumulation seem to have a strong effect on G1 arrest and the induction of cell death. Nevertheless, although this appears to be the prevalent mode of cell killing by UVB in G1-synchronized CS-B cells, it does not preclude the possibility of DNA replication also being a crucial event leading to cell-death induction in unsynchronized cells following elevated UV irradiation, since transcriptionally blocked genes may constitute important lesions, not easily bypassed by replication forks, thereby also triggering cell death [27].

**Figure 6:**
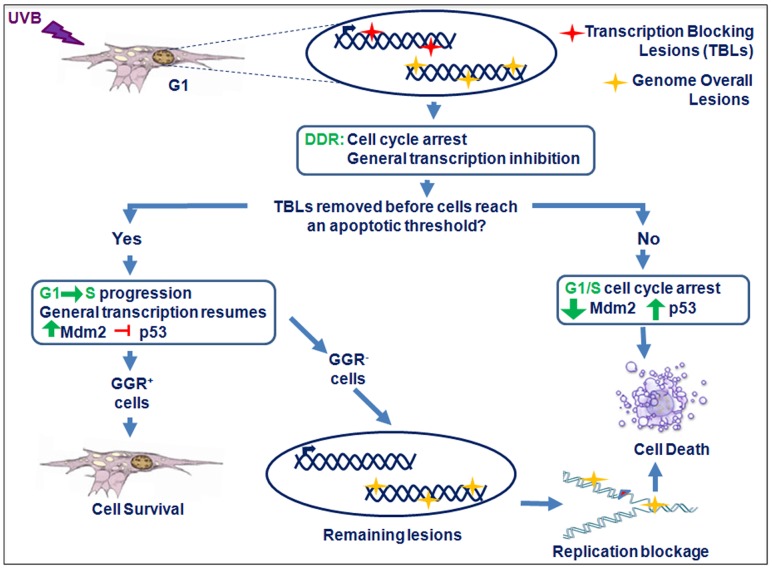
Proposed model for the different responses of human cells to UVB irradiation depending on their ability to remove DNA lesions. Upon UVB-irradiation of human fibroblasts synchronized in the G1 phase of the cell cycle, the presence of lesions on transcribed regions of the genome elicits the DNA Damage Response (DDR), comprising DNA repair, cell cycle arrest, and general transcription inhibition. In CS-B cells, the lack of TCR leads to low levels of Mdm2 and apoptosis induction. When DNA repair removes transcription blocking lesions (TBLs), Mdm2 counteracts p53 activation and cells are released to go through S-phase, and survive higher levels of lesions. In the case of XP-C (GGR^−^) fibroblasts, however non-removed lesions lead to replication blockage and cell death, even in the presence of increased levels of Mdm2.

As to XP-C cells, transcription inhibition and the accumulation of Mdm2 still occurred, but at doses similar to those for DNA repair proficient cells. Due to the excess of unrepaired lesions, the higher sensitivity of these cells to UVB irradiation was more dependent on DNA replication blockage. Therefore, both types of cell death mechanisms are potentially triggered, as represented in the schematic illustration of the proposed model on Figure 6. Transcription and replication blockages both play their part, when saturated levels of unrepaired DNA damage are encountered by the DNA processing machinery in the genome of UVB-irradiated cells.
